# The Evolution and Expression Pattern of Human Overlapping lncRNA and Protein-coding Gene Pairs

**DOI:** 10.1038/srep42775

**Published:** 2017-03-27

**Authors:** Qianqian Ning, Yixue Li, Zhen Wang, Songwen Zhou, Hong Sun, Guangjun Yu

**Affiliations:** 1 Department of Bioinformatics and Biostatistics, School of Life Sciences & Biotechnology, Shanghai Jiao Tong University, Shanghai 200240, China; 2 Shanghai Center for Bioinformation Technology, Shanghai 200235, China; 3 Key Lab of Computational Biology, CAS-MPG Partner Institute for Computational Biology, Shanghai Institutes for Biological Sciences, Chinese Academy of Sciences, Shanghai 200031, China; 4 Collaborative Innovation Center for Genetics and Development, Fudan University, Shanghai, China; 5 Medical oncology department, shanghai pulmonary hospital, cancer institute, Tongji University Medical School, Shanghai 200433, China; 6 Biomedical Information Research Center, Children’s Hospital of Shanghai, Shanghai 200062, China; 7 Children’s Hospital of Shanghai, Shanghai Jiao Tong University, Shanghai 200062, China

## Abstract

Long non-coding RNA overlapping with protein-coding gene (lncRNA-coding pair) is a special type of overlapping genes. Protein-coding overlapping genes have been well studied and increasing attention has been paid to lncRNAs. By studying lncRNA-coding pairs in human genome, we showed that lncRNA-coding pairs were more likely to be generated by overprinting and retaining genes in lncRNA-coding pairs were given higher priority than non-overlapping genes. Besides, the preference of overlapping configurations preserved during evolution was based on the origin of lncRNA-coding pairs. Further investigations showed that lncRNAs promoting the splicing of their embedded protein-coding partners was a unilateral interaction, but the existence of overlapping partners improving the gene expression was bidirectional and the effect was decreased with the increased evolutionary age of genes. Additionally, the expression of lncRNA-coding pairs showed an overall positive correlation and the expression correlation was associated with their overlapping configurations, local genomic environment and evolutionary age of genes. Comparison of the expression correlation of lncRNA-coding pairs between normal and cancer samples found that the lineage-specific pairs including old protein-coding genes may play an important role in tumorigenesis. This work presents a systematically comprehensive understanding of the evolution and the expression pattern of human lncRNA-coding pairs.

Overlapping genes were first identified in virus^1^ and subsequently found in vertebrate genomes^2^,^3^. Aside from contracting genome size, overlaps have been hypothesized to be involved in regulating gene expression at diverse levels, including transcription, mRNA splicing, transport, processing, stability and translation^4^,^5^,^6^. The transcription of antisense genes affects both the splicing and the expression of sense genes in human^7^ and the expression of overlapping genes are highly correlated^8^,^9^. A mutation in overlapping region may disrupt the function of the two genes simultaneously. Nevertheless, overlapping genes do not show higher sequence conservation compared with non-overlapping genes and the overlap structure are poorly preserved during evolution^8^,^10^,^11^.

Several hypotheses have been proposed to explain the origin of overlapping genes^11^,^12^,^13^. Generally, because of the interdependence of overlapping genes, overlapping regions are reasonably under strong selective pressure. In fact, both purifying selection and positive selection have been found in members of overlapping genes^14^,^15^,^16^, which provides evidence for the hypothesis that overlapping genes could originate via overprinting, a process generating new genes from pre-existing sequences^14^,^16^. A distinctive characteristic of overlapping genes originated from overprinting is that the new genes appear to be lineage-specific and the old partners are widespread across species^13^. Another study of overlapping genes, *ACAT2* (acetyl-CoA acetyltransferase 2) and *TCP1* (t-complex protein 1), showed that the overlap of two previously separated genes may arise during evolution through one of two ways. In one scenario, one of the genes may lose functional signals through translocation. By chance, adoption of lost signals from the new neighboring gene let this gene continue to function normally and the two genes were overlapped. Or, two fixed genes became neighboring genes through genomic rearrangement and subsequent change in the gene structure resulted in overlap^12^.

According to the coding potential of genes, overlapping genes can be categorized as coding-coding, coding-noncoding and noncoding-noncoding pairs^17^. lncRNAs are known to regulate the expression of protein-coding genes through *cis*-acting or *trans*-acting regulation mechanisms^18^,^19^,^20^,^21^. As expected for regulatory molecules, lncRNAs tend to be expressed at lower level and display higher tissue specificity than protein-coding genes^22^,^23^. Although numbers of lncRNAs are conserved across vertebrates^22^,^23^,^24^, most lncRNAs are subject to rapid turnover during evolution in terms of sequence and transcription^22^,^25^. Until now, lncRNAs overlapping with protein-coding genes have got particular attention and many studies have uncovered various mechanisms of lncRNAs regulating the expression of their protein-coding overlapping partners^19^,^26^,^27^. The dysregulation of overlapping lncRNAs also has been observed in cancer^28^,^29^,^30^ and mutated lncRNAs co-localized with protein-coding genes may act as prognostic biomarkers and therapeutic targets for cancer^30^,^31^,^32^.

Herein, we showed a systematically comprehensive understanding of the evolution and expression pattern of lncRNA-coding pairs in human genome. Through testing the origin of lncRNA-coding pairs, we observed the preference for the retention of genes in lncRNA-coding pairs during evolution. The overlapping configuration and the evolutionary age of genes were taken into account when estimating the effect of overlap on expression and co-expression of lncRNA-coding pairs. Further investigation was conducted by comparing behaviors of lncRNA-coding pairs between carcinomas and normal samples, which is indicative of the contribution of lncRNA-coding pairs to tumorigenesis.

## Results

### Overlap benefits the retention of genes

We initiated our study on the data originally produced by Necsulea *et al*.^22^. with a particular focus on human lncRNA genes overlapping with protein-coding genes. Of the total 24,793 annotated human lncRNA genes, about 29% were overlapped with protein-coding genes (Table 1) and 26% of protein-coding genes were in overlap (Supplementary Table 1).

It has been suggested that lncRNA genes evolve more rapidly than protein-coding genes^25^ and overlapping genes occur in a continuous evolutionary process^11^. We therefore asked whether the evolutionary age of genes would influence the overlap of lncRNA with protein-coding genes. In general, lncRNAs were younger than their protein-coding overlapping partners in most (86.5%) lncRNA-coding pairs. Only around one-tenth of lncRNA-coding pairs shared the same time period of origin and about 86% of pairs included old protein-coding genes originated more than 300 million years (Myr) ago (Supplementary Table 2). There were 108 clusters that lncRNAs of distinct times of origin overlapped with a single protein-coding gene. GO analysis of these protein-coding genes showed strong enrichment for terms related to the neurogenesis and hippocampus development (q value = 0.02). These lncRNAs, through successive waves of origination, may have contributed to the evolution and functional refinement of human neurons.

To address the impediment imposed by the insufficient genome annotations of some species, we integrated the human lncRNA genes into three age groups and observed that the percentage of lncRNA genes overlapping with protein-coding genes increased significantly with their evolutionary age (Table 1). The same trend was observed in protein-coding genes (Supplementary Table 1). These observations could be explained by two reasons. One is that there are selective pressures for the retention of genes in this genomic organization. The other one is that established genes are advantageous to the occurrence of overlap, indicating that lncRNA-coding pairs mainly originate from two fixed genes.

**Figure 1:**
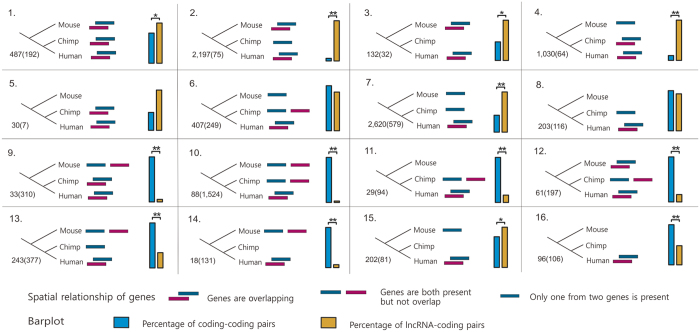
Evolutionary scenarios of human lncRNA-coding pairs and coding-coding pairs. Numbers of pairs are shown, outside the parenthesis for lncRNA-coding pairs and inside for coding-coding pairs. The bars in boxes represent the proportion of overlapping pairs with the evolutionary pattern in all corresponding pairs and asterisks indicate the statistical significance of different proportions between lncRNA-coding pairs and coding-coding pairs (one asterisk for p value < 0.05 and two for p value < 10^−5^).

We further investigated the evolutionary pattern of human overlapping genes based on comparisons with chimpanzee and mouse genomes. The evolutionary scenarios revealed that the overlap of lncRNAs and protein-coding genes occurred more likely as a result of overprinting (pattern 4, 7, 8, Fig. 1), and 49% of lncRNA-coding pairs fit exactly the hypothesis. By contrast, orthologs of coding-coding pairs frequently existed but did not overlap in the chimp and mouse (patterns 9–11, Fig. 1), which is consistent with the hypotheses that overlapping genes could be generated by genomic rearrangement and adoption of signals from neighboring genes or by change in gene structure. There were only 150 (2%) lncRNA-coding pairs fitting this pattern, indicating that the higher percentage of overlapping genes in old group is mainly caused by the evolutionary advantage for the retention of genes in overlap.

### The preference of overlapping configurations based on the origin is preserved through evolution

**Figure 2:**
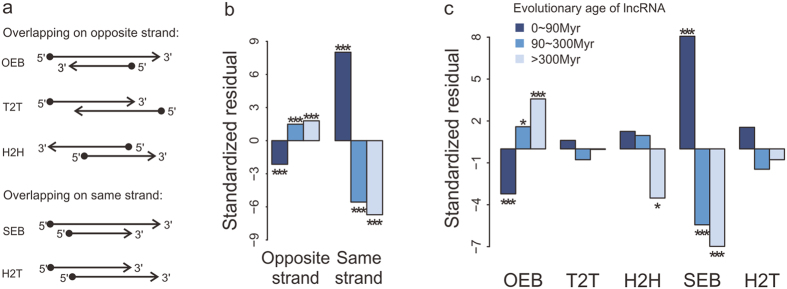
Overlapping configuration preference of human lncRNA-coding pairs. (**a**) Schematic representation of lncRNA-coding pairs, according to the orientation of the overlapping genes. Arrows indicate the orientation directions of genes. (**b**,**c**) The preference of lncRNA-coding pairs in overlapping strands (**b**) or overlapping configurations (**c**), according to the evolutionary age of human lncRNA genes. The standardized residuals were calculated in a 2 × 2 contingency table and the asterisks on the bar stand for the statistical significances of Chi square test: one for p < 2.5 × 10^−3^, two for p < 10^−5^ and three for p < 10^−10^.

To test whether the overlapping configuration would affect the evolution of lncRNA-coding pairs, we first classified them into 5 groups depending on the orientation of transcripts involved. Pairs overlapped on the opposite strand were classified as: head-to-head (H2H, 5′-regions overlap), tail-to-tail (T2T, 3′-regions overlap) and embedded (OEB) pairs. And pairs overlapped on the same strand were classified as: head-to-tail (H2T, 5′-region overlap with 3′-region) and embedded (SEB) pairs (Fig. 2a).

Generally, overlaps on the opposite strand amounted to almost ninety-three percent and embedded pairs were much more than partially overlapping genes (Supplementary Table 3). Considering the evolutionary age of lncRNAs, old lncRNA genes were more likely to be embedded within protein-coding genes on the opposite strand but less on the same strand. An exactly opposite tendency of young lncRNA genes was observed (Fig. 2b,c). Additionally, old lncRNA genes showed lower preference for H2H compared with young lncRNA genes (Fig. 2c). These observations suggest that lncRNA-coding pairs express a strong preference to be embedded and different-strand overlaps. Theoretically, activating two overlapping transcriptional units at the same time is unlikely, which would result in transcriptional interference^6^. And we found that protein-coding genes overlapped with lncRNAs on the same strand had significantly lower expression level than on the opposite strand (Wilcoxon signed-rank test, p value = 2.4 × 10^−3^), with a median RPKM of 10.7 on the same strand and 12.7 on the opposite strand, respectively. Therefore, lncRNAs overlapped protein-coding genes on the same strand are less desirable. Since few lncRNA-coding pairs originate from genomic rearrangement and change in gene structure, the main sources of partially overlapping genes, embedded pairs are easy to be found in lncRNA-coding pairs.

We then assessed the evolutionary conservation of lncRNA-coding pairs in the sense of genomic structure and overlapping configuration. Of the total 7,876 human lncRNA-coding pairs, only orthologs of 487 pairs involved in overlaps both in the chimpanzee and mouse genome (Fig. 1, Supplementary File 1). But the composition of overlapping configurations in the conserved pairs was not significantly different from the total pairs (Supplementary Table 3), suggesting that the overlapping configuration is not related to the evolution of overlap. All the above observations demonstrate that the origin of overlapping genes confines the preference of overlapping configurations which is preserved during the evolution of overlapping genes.

### The alternative splicing pattern of lncRNA-coding pairs is related to the overlapping configuration

It has been reported that a number of lncRNA genes possess the canonical splice site consensus motifs^33^ and the antisense expression can affect mRNA splicing of sense genes^7^. To further explore whether the overlapping configuration would affect the alternative splicing pattern of lncRNA-coding pairs, we downloaded the alternative transcript annotations of human lncRNA and protein-coding genes from Ensembl^34^. Around 26% of the annotated human lncRNAs produced alternative transcripts, and 48% of them overlapped with protein-coding gene(s) (Supplementary Table 4). According to the number of alternative transcripts annotated for the lncRNA and protein-coding gene, the alternative splicing patterns of lncRNA-coding pairs were classified as single-to-single (SS), single-to-multiple (SM), multiple-to-single (MS) or multiple-to-multiple (MM) patterns (the first letter was representative for the lncRNA gene and the second for the protein-coding gene).

**Figure 3:**
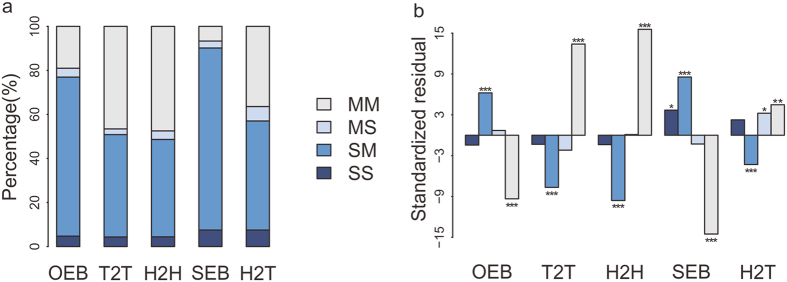
The relationship between alternative splicing pattern and overlapping configuration of human lncRNA-coding pairs. (**a**) Composition of alternative splicing patterns of human lncRNA-coding pairs in each overlapping configuration. (**b**) The preference of overlapping configurations for each alternative splicing pattern. The standardized residuals were calculated in a 2 × 2 contingency table and the asterisks on the bar stand for the statistical significances of Chi square test: one for p < 2.5 × 10^−3^, two for p < 10^−5^ and three for p < 10^−10^.

There was a clear association between the alternative splicing pattern and the overlapping configuration of lncRNA-coding pairs (Supplementary Table 5). As shown in Fig. 3b, those lncRNA-coding pairs with SS pattern were more likely to be embedded on the same strand and more SM pairs were observed with embedded form and less with partially overlapping form. For lncRNA-coding pairs with MS pattern, the H2T configuration was preferred over other configurations and those MM pairs showed right opposite preference with SM pairs, more with partially overlapping form and less with embedded form. These observations imply that the alternative splicing pattern of lncRNA-coding pairs is related to the type of overlapping configuration.

Antisense lncRNA could affect the alternative splicing of sense protein-coding gene^35^ and overlap regions are potential hotspots for the splicing regulation^7^. Consistent with that, there were only a few lncRNA-coding pairs with SS pattern and the majority of pairs were overlaps with SM and MM patterns (Fig. 3a). Furthermore, more protein-coding genes generating multiple products overlapped with lncRNAs, but lncRNAs did not (Supplementary Table 4). It reveals that the antisense transcription-mediated mechanism of splicing regulation is a unilateral interaction.

### Overlapping genes have higher expression level and tissue specificity

**Figure 4:**
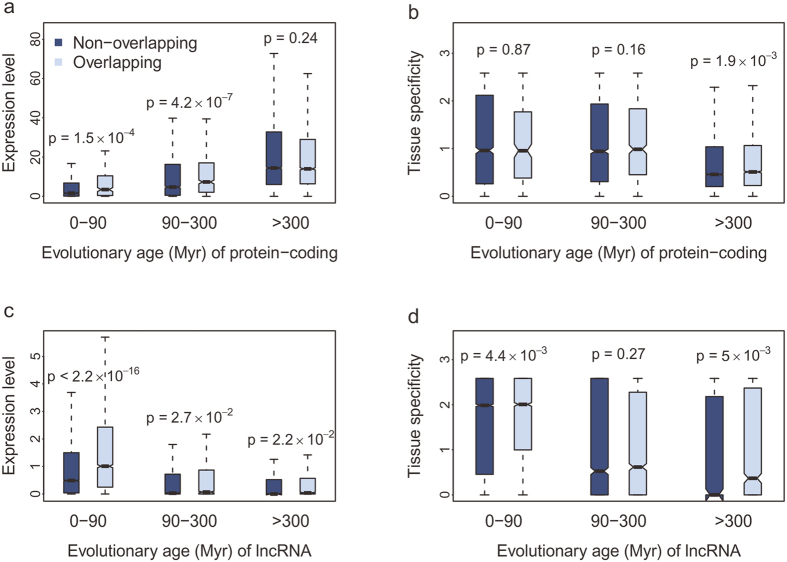
Higher expression level and tissue specificity of lncRNA-coding pairs. (**a,c**) The maximum expression level (RPKM) of protein-coding (**a**) or lncRNA (**c**) genes by evolutionary age. (**b,d**) The tissue specificity of protein-coding (**b**) or lncRNA (**d**) genes by evolutionary age.

The antisense expression has been reported to affect the expression of sense genes^7^, then the potential regulatory interactions mediated by the genomic organization was assessed. For the young protein-coding genes (age < 90 Myr), the expression levels of overlapping genes were significantly higher than that of non-overlapping ones and the gap narrowed with the increase of evolutionary age (Fig. 4a). And for lncRNAs, the expression levels of overlapping genes were higher than non-overlapping genes in all groups (Fig. 4c). The data suggest that the genomic structure may benefit the expression of lncRNA-coding pairs and the effect on genes is age-specific. Additionally, in old group, both lncRNAs and protein-coding genes in lncRNA-coding pairs had higher tissue specificity than non-overlapping genes (Fig. 4b,d), which indicates that overlap may diversify the function of genes through confining the expression spectrum of overlapping genes. Taken together, the genomic organization improves the expression level and is conducive to confining the expression breadth of genes. The effect of overlap on gene expression is more complex in chimp and mouse. Similarly, for protein-coding genes, the existence of overlapping partners increased the expression level of young genes and the tissue specificity of old genes. But the effect of overlap on lncRNAs was a little different, where the tissue specificity was lower than non-overlapping genes (Supplementary Figs 1 and 2).

**Figure 5:**
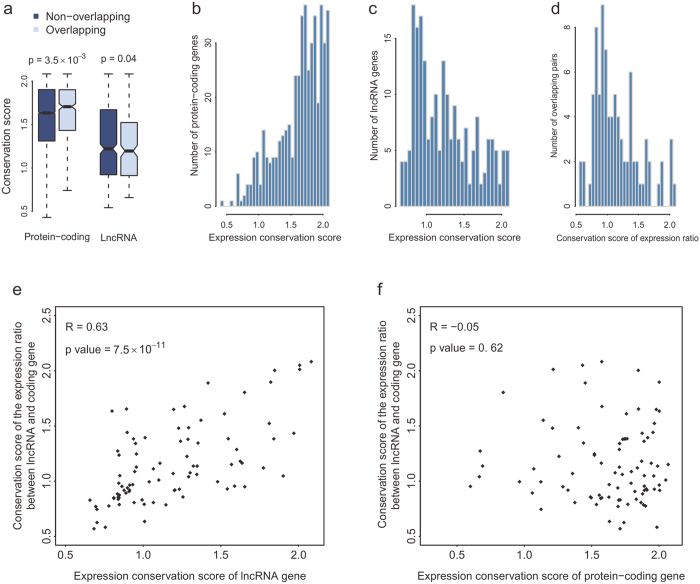
Expression conservation of lncRNA-coding pairs. (**a**) Expression conservation score of protein-coding and lncRNA genes. The conservation score ranges from 0 to 2 and values close to 2 represent highly conserved expression. (**b**,**c**) Distribution of expression conservation score of the protein-coding (**b**) or lncRNA (**c**) genes in lncRNA-coding pairs. (**d**) The conservation score of expression ratio of lncRNA-coding pairs. The expression ratio was calculated by the expression of lncRNA gene over its protein-coding overlapping partner. (**e,f**) The correlation between the expression ratio conservation and the expression conservation of lncRNA (**e**) or protein-coding (**f**) genes.

To explore the effect of overlap on the expression conservation of genes, the conservation score of gene expression was calculated. The expression of protein-coding genes in lncRNA-coding pairs was more conserved than non-overlapping genes, whereas lncRNAs in overlap had lower expression conservation than non-overlapping genes (Fig. 5a), suggesting that the genomic structure promotes the expression conservation of protein-coding genes rather than lncRNAs. For the 487 conserved lncRNA-coding pairs, the expression conservation scores of protein-coding genes were skewed towards the highest value (Fig. 5b), while the score of lncRNA genes showed a broader distribution (Fig. 5c), which is consistent with the finding that lncRNAs have more rapid transcriptional turnover than protein-coding genes^22^,^25^. The conservation scores of the expression ratios of lncRNAs over their protein-coding overlapping partners were also calculated and the value was scattered as lncRNAs (Fig. 5d), suggestive of the barely conserved coordinated expression of the lncRNA-coding pairs. The conservation degree of the expression ratios was significantly correlated with lncRNAs (Fig. 5e), whereas no significant correlation was observed when considering protein-coding genes (Fig. 5f), which confirms the regulatory role of lncRNAs.

### Genes in lncRNA-coding pairs are widely co-expressed

**Figure 6:**
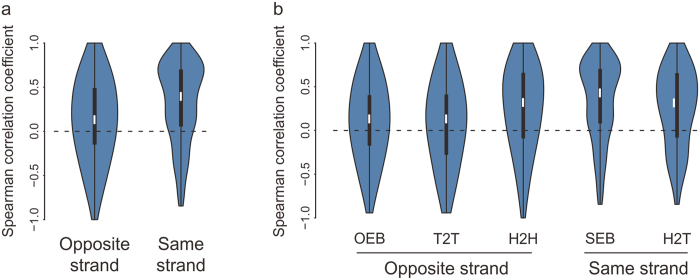
Widespread expression correlation of lncRNA-coding pairs. (**a**,**b**) Distribution of Spearman correlation coefficient between lncRNA and its protein-coding overlapping partner by overlapping strand (**a**) or overlapping configuration (**b**). Vioplot also displays the full distribution of data, not only the summary statistics.

Overlapping genes are known to couple gene expression^9^. We thus tested the expression correlation of lncRNA-coding pairs and observed that the expression of lncRNA-coding pairs showed an overall positive correlation, with a median Spearman correlation coefficient of 0.21 for different-strand overlaps and 0.41 for same-strand overlaps, respectively (Fig. 6a). Among all the lncRNA-coding pairs, SEB pairs under similar local chromatin environment displayed the highest expression correlation (median R = 0.43). And the expression of H2H pairs showed the strongest positive correlation (median R = 0.31) in pairs overlapped on the opposite strand (Fig. 6b).

It has been well studied that the bidirectional-like promoters contribute to the coordinated expression of H2H pairs^9^,^36^. To assess the effect of bidirectional promoters, we roughly searched for identical transcription factor binding sites (TFBSs) within the 1-kb upstream genomic regions of the two transcriptional start sites. More H2H pairs contained identical TFBS(s) within the two independent upstream regions when compared with the other two overlapping configurations on the opposite strand (Supplementary Table 6) and only H2H pairs with identical TFBS(s) had higher expression correlation than pairs with no identical TFBS (Supplementary Fig. 3), suggesting that the expression of H2H pairs is likely coordinated by similar regulatory sequences.

Previous study has proved that lncRNAs and nearby protein-coding genes are co-expressed^37^, then we grouped lncRNA-coding pairs with neighboring pair(s) within a 40-Kb genomic distance into blocks to estimate the effect of local genomic environment. Around 54% of lncRNA-coding pairs were falling into blocks with more than one pair (Supplementary File 2). The expression correlation coefficients of lncRNA-coding pairs were less dispersed among the block pairs (mean SD = 0.24) than the corresponding individual pairs (SD = 0.44; Student test for the mean difference, p value < 2.2 × 10^−16^).

Taking the evolutionary age of genes into account, the expression correlation of lncRNA-coding pairs was significantly weakened with the increased evolutionary age of protein-coding genes, but not with lncRNAs. Young protein-coding genes originated less than 90 Myr ago had a relatively stronger correlation (median R = 0.39) than old protein-coding genes (median R = 0.13) with their lncRNA overlapping partners (Supplementary Fig. 4). It could partially be explained by the fact that old protein-coding genes are required for the maintenance of the cell fundamental functions and their expression should remain a relatively stable level. These results together suggest that the overlapping configuration, local genomic environment and evolutionary age of genes have an influence on the expression correlation of lncRNA-coding pairs.

### Signatures of co-expression of lncRNAs-coding pairs for carcinoma

**Figure 7:**
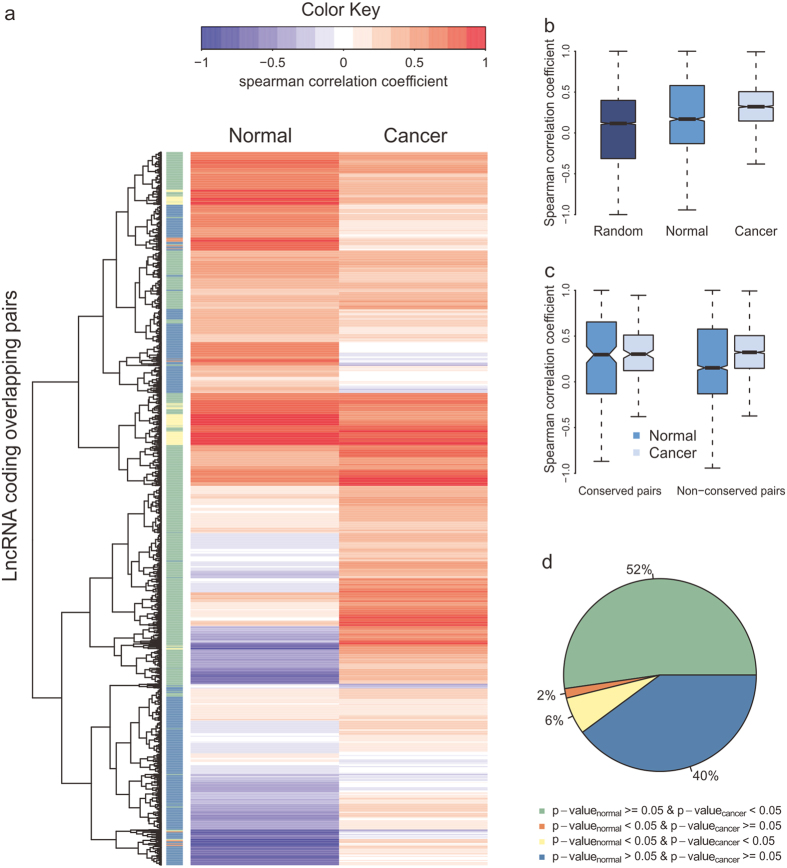
Expression correlation patterns of lncRNA-coding pairs in normal and cancer. (**a**) Heatmap of Spearman correlation coefficient of lncRNA-coding pairs in normal and cancer. (**b**) Boxplot of Spearman correlation coefficient of random pairs or overall pairs in normal or cancer. (**c**) The Spearman correlation coefficient of conserved pairs and non-conserved pairs in normal and cancer. (**d**) Composition of lncRNA-coding pairs based on the significance of expression correlation. The P_normal_ represents the p value of expression correlation of lncRNA-coding pairs in normal and P_cancer_ indicates the p value in cancer.

Potential lncRNA-disease associations have been identified by computational models^38^,^39^,^40^,^41^,^42^ and aberrant expression of antisense RNA may contribute to cancers^43^,^44^,^45^. As co-expression between overlapping partners has been frequently reported^46^,^47^, we investigated whether there existed any signature in dysregulated coordinated expression of lncRNA-coding pairs using an RNA sequencing dataset of 369 cancer samples^9^. Genes with low level of expression were excluded and 2,122 human lncRNA-coding pairs (Supplementary File 3) were left for the further analysis. The patterns of the expression correlation of lncRNA-coding pairs were distinct in normal and cancer (Fig. 7a) and the lncRNA-coding pairs displayed significantly higher correlation in cancer (Fig. 7b). Around 52% of lncRNA-coding pairs were only significantly correlated in cancer and only about two percent showed an opposite tendency. Six percent of lncRNA-coding pairs were correlated in both normal and cancer samples (Fig. 7d).

The expression of non-conserved or lineage-specific lncRNA-coding pairs had significantly higher correlation in cancer, while pairs conserved in human, chimp and mouse genomes did not (Fig. 7c). For the three age groups of protein-coding genes, only the expression of old genes showed significantly stronger correlation with their partners in cancer (median R = 0.32) than in normal (median R = 0.14, Supplementary Fig. 5a). The possible reasons may be that a small portion of pairs included protein-coding genes originated less than 300 Myr ago and those protein-coding genes showed no significant functional enrichment, as well as genes in conserved pairs (Supplementary Figs 6 and 7). In contrast, old genes in non-conserved pairs are functional in various processes, like development and also cell-cell signal pathway (Supplementary File 5). The regulatory phenotypic profiles as a part of cancer hallmark network framework would lead to clinical phenotype^48^. Therefore, we could speculate that the altered expression correlation pattern of lineage-specific lncRNA-coding pairs, especially pairs containing protein-coding genes originated more than 300 Myr ago, may play an important role in tumorigenesis. But for coding-coding pairs, the expression correlation was stronger in cancer among all age groups and that of conserved pairs also showed significant increase in cancer (Supplementary Fig. 5c,d).

Several lncRNA-coding pairs with exactly opposite types of correlational relationship in cancer and normal were identified (Supplementary File 4). Interestingly, the expression of *SAMSN1* and *SAMSN1 antisense RNA 1* were negatively correlated in normal (R = −0.85, p = 0.03), but positively correlated in cancer (R = 0.70, p = 4.2 × 10^−9^), implicating the absence of the suppression of *SAMAN1* by lncRNA in cancer. *SAMSN1* is predominantly expressed in immune tissues and hematopoietic cells, with lower expression in heart, brain, placenta, and lung^49^. Since the expression data of cancer we used were mainly from lung, prostate, ovary and brian, it was reasonable that the lncRNA-coding pair was positively correlated in cancer. Previous studies have testified that the *SAMSN1* expression is low or absent in human myeloma cell lines^50^ and the absence of *SAMSN1* contributes to multiple myeloma progression^51^. But the *SAMSN1* is over-expressed in glioma and the high expression of *SAMSN1* is a significant risk factor for the progression of glioblastoma multiforme. Thus the altered correlational relationship of *SAMAN1* and *SAMSN1 antisense RNA 1* may serve as a biomarker for the prognosis and therapy of cancer.

## Discussion

Genes in lncRNA-coding pairs are more likely to be retained throughout evolution. Protein-coding overlapping genes originated through overprinting are constrained to the 123:132 phase which ensures the least mutual constraint on both protein sequences^15^. Since lncRNA genes have no reading frame, evolve rapidly and are less conserved than protein-coding genes in terms of sequence^25^, it is more likely for lncRNA to be generated from an pre-existing coding sequence. Indeed, nearly half of lncRNA-coding pairs were found to be generated by overprinting and few pairs were from changing the spatial relationship of two separated genes. However, most human coding-coding pairs were the results of genomic rearrangement or elongation of two genes, similar with the study of Fukuda *et al*.^52^. Considering the origin of overlaps, the trend that the percentage of genes in overlap increases with the evolutionary age declares that overlap is advantageous to the retention of genes throughout evolution. Furthermore, the observation that protein-coding genes overlapped with lncRNAs originated from different time periods, could play a role in establishing or maintaining cellular diversity and may contribute to the species diversification.

Overlapping configurations are mainly affected by the origin of overlapping genes. Partially overlapping genes usually arise from genomic rearrangement or elongation of two fixed genes and introns as a valuable evolutionary source for overprinting^13^, hints that overlapping genes originated from this way may occur as embedded pairs. Since more lncRNA-coding pairs were generated by overprinting and few from the change of the spatial relationship of two fixed genes, higher percentage of embedded pairs was observed. Also, the different-strand overlaps accounted for the majority of lncRNA-coding pairs because of the transcription interference of overlaps on the same strand. But there was no difference between the overlapping configuration compositions of the conserved and all lncRNA-coding pairs. These results suggest that the overlapping configuration only depends on the origin of overlapping genes and the subsequent evolution has no influence on it.

Overlap enhances the expression level and tissue specificity of genes in lncRNA-coding pairs and these effects are age-specific. The expression level of genes in lncRNA-coding pairs was higher than that of non-overlapping genes and the increase was more obvious in young group. By contrast, the tissue specificity of overlapping genes was remarkably improved in old group. These may give us a clue that the existence of overlapping partners adjusts the expression level and expression breadth of genes in lncRNA-coding pairs and these effects differ at different age groups. Considering the expression conservation of genes, the expression of protein-coding genes was much more conserved than lncRNA genes. However, the overlap structure only improved the expression conservation of protein-coding genes not lncRNA genes. Comparisons of the expression ratio conservation with the expression conservation of lncRNA and protein-coding genes in lncRNA-coding pairs confirmed the regulatory role of lncRNAs.

Expression correlation is a predominant characteristic of overlapping genes^8^,^9^ and overlapping configurations, local genomic environment and the evolutionary age of genes are important factors influencing this correlation. SEB pairs, under common regulatory system, showed the highest expression correlation and H2H pairs had higher correlation than other different-strand overlaps. It has been reported that bidirectional promoter coordinates the expression of sense gene and antisense lncRNA^53^, which would be the reason for the stronger correlation of H2H pairs. The deviation of expression correlation coefficient of individual lncRNA-coding pairs was significantly larger than those in blocks, suggestive of the important role of the local genomic environment. Then, taking the evolutionary age of genes into account, newly evolved protein-coding genes had higher expression correlation with their lncRNA partners than old genes. It indicates that young protein-coding genes are more flexible than old genes whose expression should be maintained at a relatively stable level.

The expression correlation pattern of lncRNA-coding pairs was altered in cancer, which may contribute to tumorigenesis. Although the expression correlation of lncRNA-coding pairs was higher in caner, the conserved pairs and pairs including protein-coding genes originated less than 300 Myr ago had no significant difference between normal and cancer, which is different from coding-coding pairs. For lncRNA-coding pairs, old protein-coding genes in non-conserved pairs showed functional enrichment in terms of development and morphogenesis, which remind us that the aberrant regulatory phenotype of those pairs play an important role in carcinogenesis. Additionally, pairs both correlated in normal and cancer tissues with opposite type of correlational relationship may promote pathogenesis of cancer.

Through detecting the orthologs of human lncRNA-coding pairs in chimp and mouse genomes, initial attempts were made to investigate the origin and evolution of lncRNA-coding pairs. We are well aware that the study on few genomes may lead to biased conclusions, so further comparative studies about lncRNA-coding pairs based on more well-annotated genomes are necessary. However, our study did present a relatively comprehensive understanding of the evolution and expression pattern of lncRNA-coding pairs.

## Data and Methods

### Data

The annotations of lncRNA and protein-coding genes used to identify lncRNA-coding pairs, the orthology information of lncRNA genes, the strand-specific and non-strand-specific expression data for expression correlation, tissue specificity and expression conservation were obtained from Necsulea *et al*.^22^. The alternative transcripts information of human lncRNA and protein-coding genes (Ensembl v85) was downloaded from Ensembl Genome Browser database (http://www.ensembl.org/index.html)^34^. Considering the genome annotation version used by Necsulea *et al*. to annotate lncRNA and protein-coding genes, the conserved transcription factors binding sites (TFBSs) based on GRCh37 were downloaded from UCSC Genome Browser database (http://genome.ucsc.edu/) by Table Browser^54^. In addition, the expression data of 369 carcinoma samples were obtained from Balbin *et al*.^9^.

### Identification of lncRNA-coding pairs

The lncRNA-coding pairs were identified under the criteria that two transcripts shared at least one nucleotide and only the longest form of alternative splicing was considered. To estimate the effect of local genomic environment on the co-expression of lncRNA-coding pairs, all pairs were grouped into distinct blocks. If the lncRNA-coding pair has neighboring pair(s) within a 40-Kb genomic distance, these pairs were considered as a block. All pairs in this block were then detected until no pairs had neighboring pair within a 40-Kb genomic region.

### Identification of orthologous genes and the evolutionary age of human protein-coding genes

Based on the homology information of any two genomes provided in InParanoid8^55^ and Ensembl^34^, the orthology inference was done. Orthologous genes of human protein-coding genes were first selected from InParanoid8 (http://inparanoid.sbc.su.se/download/)^55^ by inparalog score equal to one in 9 species: chimpanzee, gorilla, orangutan, macaque, mouse, opossum, platypus, chicken and *Xenopus*. The threshold used to select orthologous genes from Ensembl is the identity great than 55%, a value below which were the 5% of the identity scores between orthologous genes from InParanoid8 and human genes. The union set of orthologs from InParanoid8 and Ensembl in given genomes was then used in subsequent analysis. Briefly, the minimum evolutionary age of protein-coding genes was inferred based on the presence of orthologs without taking transcription evidence into account, as lncRNA genes inferred by Necsulea *et al*.^22^.

### Construction of evolutionary scenarios of human lncRNA-coding and coding-coding pairs

For each of lncRNA-coding or coding-coding pairs in human genome, spatial relationships of their orthologs in chimp and mouse genomes were checked based on the orthology inferred above. Since the relationship between human protein-coding genes and their orthologous genes in other species was not a simple one-to-one relationship, each of the orthologous genes was checked for overlaps in corresponding genome. Based on the presence and spatial relationships of orthologs in given genomes, the evolutionary scenarios of human overlapping genes were classified into sixteen patterns as in Fig. 1.

### Calculation of tissue specificity

To detect the expression specificity of lncRNA and protein-coding genes across tissues, the mean expression levels of genes in each tissue were obtained. We used the following algorithm proposed by Landgraf *et al*.^56^ to calculate the tissue specificity of the expression of lncRNA and protein-coding genes:


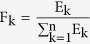



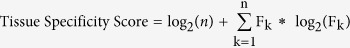


where E_K_ was the mean expression level of a gene in tissue k, and n was the number of tissue types.

### Expression conservation score

As Liao *et al*. presented^57^, we extracted non-strand-specific expression data from common tissues of two species and normalized by their relative abundance (RA):


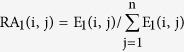



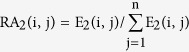


where n meant the number of tissue types, and E_1_ (i, j) was the mean expression level of gene i in tissue j of species 1. The expression conservation score of gene i between species 1 and 2:


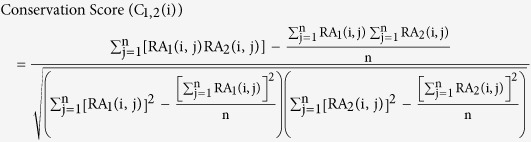


Then the conservation score of gene i among three species:


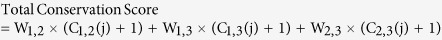


where W_1,2_ was the phylogenetic distance between species 1 and 2. Considering that the conservation score ranges from -1 to 1, we added 1 to adjust the conservation score to positive when weighted by the pair-wise phylogenetic distance. The conservation score of expression ratio was also calculated as above.

## Additional Information

**How to cite this article**: Ning, Q. *et al*. The Evolution and Expression Pattern of Human Overlapping lncRNA and Protein-coding Gene Pairs. *Sci. Rep.* **7**, 42775; doi: 10.1038/srep42775 (2017).

**Publisher's note:** Springer Nature remains neutral with regard to jurisdictional claims in published maps and institutional affiliations.

## Supplementary Material

Supplementary Information

Supplementary Dataset 1

Supplementary Dataset 2

Supplementary Dataset 3

Supplementary Dataset 4

Supplementary Dataset 5

## Figures and Tables

**Table 1:** Preference of overlap in old group of lncRNA genes.

Age (Myr)	Overlapping		Non-overlapping		Total
	Obs. (%)	Exp.	Obs.	Exp.	
0~90	3844 (21.3)	5271	14168	12741	18012
90~300	2194 (44.6)	1439	2725	3480	4919
>300	1217 (65.4)	545	645	1317	1862
Total	7255 (29.3)		17538		24793

Chi square test was used to test for statistical significance: χ^2^ = 2,277, p value < 2.2 × 10^−16^. The percentage in the parenthesis was calculated as the number of genes in lncRNA-coding pairs divided by the total number of genes in each age group.

## References

[b1] Barrell B. G., Air G. M. & Hutchison C. A. 3rd. Overlapping genes in bacteriophage phiX174. Nature 264, 34–41 (1976). 1004533 10.1038/264034a0

[b2] Spencer C. A., Gietz R. D. & Hodgetts R. B. Overlapping transcription units in the dopa decarboxylase region of Drosophila. Nature 322, 279–281, doi: 10.1038/322279a0 (1986). 2874495

[b3] Henikoff S., Keene M. A., Fechtel K. & Fristrom J. W. Gene within a gene: nested Drosophila genes encode unrelated proteins on opposite DNA strands. Cell 44, 33–42 (1986). 3079672 10.1016/0092-8674(86)90482-4

[b4] Johnson Z. I. & Chisholm S. W. Properties of overlapping genes are conserved across microbial genomes. Genome research 14, 2268–2272, doi: 10.1101/gr.2433104 (2004). 15520290 PMC525685

[b5] Krakauer D. C. Stability and evolution of overlapping genes. Evolution; international journal of organic evolution 54, 731–739 (2000). 10937248 10.1111/j.0014-3820.2000.tb00075.x

[b6] Makalowska I., Lin C. F. & Makalowski W. Overlapping genes in vertebrate genomes. Computational biology and chemistry 29, 1–12, doi: 10.1016/j.compbiolchem.2004.12.006 (2005). 15680581

[b7] Morrissy A. S., Griffith M. & Marra M. A. Extensive relationship between antisense transcription and alternative splicing in the human genome. Genome research 21, 1203–1212, doi: 10.1101/gr.113431.110 (2011). 21719572 PMC3149488

[b8] Ho M. R., Tsai K. W. & Lin W. C. A unified framework of overlapping genes: towards the origination and endogenic regulation. Genomics 100, 231–239, doi: 10.1016/j.ygeno.2012.06.011 (2012). 22766524

[b9] Balbin O. A. . The landscape of antisense gene expression in human cancers. Genome research 25, 1068–1079, doi: 10.1101/gr.180596.114 (2015). 26063736 PMC4484389

[b10] Veeramachaneni V., Makalowski W., Galdzicki M., Sood R. & Makalowska I. Mammalian overlapping genes: the comparative perspective. Genome research 14, 280–286, doi: 10.1101/gr.1590904 (2004). 14762064 PMC327103

[b11] Makalowska I., Lin C. F. & Hernandez K. Birth and death of gene overlaps in vertebrates. BMC evolutionary biology 7, 193, doi: 10.1186/1471-2148-7-193 (2007). 17939861 PMC2151771

[b12] Shintani S., O’HUigin C., Toyosawa S., Michalova V. & Klein J. Origin of gene overlap: the case of TCP1 and ACAT2. Genetics 152, 743–754 (1999). 10353914 10.1093/genetics/152.2.743 PMC1460620

[b13] Keese P. K. & Gibbs A. Origins of genes: “big bang” or continuous creation? Proceedings of the National Academy of Sciences of the United States of America 89, 9489–9493 (1992). 1329098 10.1073/pnas.89.20.9489 PMC50157

[b14] Pavesi A. Origin and evolution of overlapping genes in the family Microviridae. The Journal of general virology 87, 1013–1017, doi: 10.1099/vir.0.81375-0 (2006). 16528052

[b15] Rogozin I. B. . Purifying and directional selection in overlapping prokaryotic genes. Trends in genetics : TIG 18, 228–232 (2002). 12047938 10.1016/s0168-9525(02)02649-5

[b16] Sabath N., Wagner A. & Karlin D. Evolution of viral proteins originated de novo by overprinting. Molecular biology and evolution 29, 3767–3780, doi: 10.1093/molbev/mss179 (2012). 22821011 PMC3494269

[b17] Yin Y. . antiCODE: a natural sense-antisense transcripts database. BMC Bioinformatics 8, 319, doi: 10.1186/1471-2105-8-319 (2007). 17760969 PMC1997216

[b18] Ponting C. P., Oliver P. L. & Reik W. Evolution and functions of long noncoding RNAs. Cell 136, 629–641, doi: 10.1016/j.cell.2009.02.006 (2009). 19239885

[b19] Geisler S. & Coller J. RNA in unexpected places: long non-coding RNA functions in diverse cellular contexts. Nature reviews. Molecular cell biology 14, 699–712, doi: 10.1038/nrm3679 (2013). 24105322 PMC4852478

[b20] Guttman M. . Chromatin signature reveals over a thousand highly conserved large non-coding RNAs in mammals. Nature 458, 223–227, doi: 10.1038/nature07672 (2009). 19182780 PMC2754849

[b21] Fatica A. & Bozzoni I. Long non-coding RNAs: new players in cell differentiation and development. Nature reviews. Genetics 15, 7–21, doi: 10.1038/nrg3606 (2014). 24296535

[b22] Necsulea A. . The evolution of lncRNA repertoires and expression patterns in tetrapods. Nature 505, 635–640, doi: 10.1038/nature12943 (2014). 24463510

[b23] Washietl S., Kellis M. & Garber M. Evolutionary dynamics and tissue specificity of human long noncoding RNAs in six mammals. Genome research 24, 616–628, doi: 10.1101/gr.165035.113 (2014). 24429298 PMC3975061

[b24] Ulitsky I., Shkumatava A., Jan C. H., Sive H. & Bartel D. P. Conserved function of lincRNAs in vertebrate embryonic development despite rapid sequence evolution. Cell 147, 1537–1550, doi: 10.1016/j.cell.2011.11.055 (2011). 22196729 PMC3376356

[b25] Kutter C. . Rapid turnover of long noncoding RNAs and the evolution of gene expression. PLoS genetics 8, e1002841, doi: 10.1371/journal.pgen.1002841 (2012). 22844254 PMC3406015

[b26] Rinn J. L. & Chang H. Y. Genome regulation by long noncoding RNAs. Annual review of biochemistry 81, 145–166, doi: 10.1146/annurev-biochem-051410-092902 (2012). PMC3858397 22663078

[b27] Lee J. T. Epigenetic regulation by long noncoding RNAs. Science 338, 1435–1439, doi: 10.1126/science.1231776 (2012). 23239728

[b28] Maruyama R. . Altered antisense-to-sense transcript ratios in breast cancer. Proceedings of the National Academy of Sciences of the United States of America 109, 2820–2824, doi: 10.1073/pnas.1010559107 (2012). 21098291 PMC3286925

[b29] Gupta R. A. . Long non-coding RNA HOTAIR reprograms chromatin state to promote cancer metastasis. Nature 464, 1071–1076, doi: 10.1038/nature08975 (2010). 20393566 PMC3049919

[b30] Salameh A. . PRUNE2 is a human prostate cancer suppressor regulated by the intronic long noncoding RNA PCA3. Proceedings of the National Academy of Sciences of the United States of America 112, 8403–8408, doi: 10.1073/pnas.1507882112 (2015). 26080435 PMC4500257

[b31] Du M. . The association analysis of lncRNA HOTAIR genetic variants and gastric cancer risk in a Chinese population. Oncotarget 6, 31255–31262, doi: 10.18632/oncotarget.5158 (2015). 26384301 PMC4741602

[b32] Wang Q. . A novel cell cycle-associated lncRNA, HOXA11-AS, is transcribed from the 5-prime end of the HOXA transcript and is a biomarker of progression in glioma. Cancer letters 373, 251–259, doi: 10.1016/j.canlet.2016.01.039 (2016). 26828136

[b33] Ponjavic J., Ponting C. P. & Lunter G. Functionality or transcriptional noise? Evidence for selection within long noncoding RNAs. Genome research 17, 556–565, doi: 10.1101/gr.6036807 (2007). 17387145 PMC1855172

[b34] Yates A. . Ensembl 2016. Nucleic Acids Res 44, D710–716, doi: 10.1093/nar/gkv1157 (2016). 26687719 PMC4702834

[b35] Gonzalez I. . A lncRNA regulates alternative splicing via establishment of a splicing-specific chromatin signature. Nature structural & molecular biology 22, 370–376, doi: 10.1038/nsmb.3005 (2015). PMC6322542 25849144

[b36] Trinklein N. D. . An abundance of bidirectional promoters in the human genome. Genome research 14, 62–66, doi: 10.1101/gr.1982804 (2004). 14707170 PMC314279

[b37] Ponjavic J., Oliver P. L., Lunter G. & Ponting C. P. Genomic and transcriptional co-localization of protein-coding and long non-coding RNA pairs in the developing brain. PLoS genetics 5, e1000617, doi: 10.1371/journal.pgen.1000617 (2009). 19696892 PMC2722021

[b38] Chen X., Huang Y. A., Wang X. S., You Z. H. & Chan K. C. FMLNCSIM: fuzzy measure-based lncRNA functional similarity calculation model. Oncotarget, doi: 10.18632/oncotarget.10008 (2016). PMC5216773 27322210

[b39] Huang Y. A., Chen X., You Z. H., Huang D. S. & Chan K. C. ILNCSIM: improved lncRNA functional similarity calculation model. Oncotarget 7, 25902–25914, doi: 10.18632/oncotarget.8296 (2016). 27028993 PMC5041953

[b40] Chen X., You Z. H., Yan G. Y. & Gong D. W. IRWRLDA: improved random walk with restart for lncRNA-disease association prediction. Oncotarget, doi: 10.18632/oncotarget.11141 (2016). PMC5295400 27517318

[b41] Chen X., Yan C. C., Zhang X. & You Z. H. Long non-coding RNAs and complex diseases: from experimental results to computational models. Brief Bioinform, doi: 10.1093/bib/bbw060 (2016). PMC5862301 27345524

[b42] Chen X. & Yan G. Y. Novel human lncRNA-disease association inference based on lncRNA expression profiles. Bioinformatics 29, 2617–2624, doi: 10.1093/bioinformatics/btt426 (2013). 24002109

[b43] Kim K. . HOTAIR is a negative prognostic factor and exhibits pro-oncogenic activity in pancreatic cancer. Oncogene 32, 1616–1625, doi: 10.1038/onc.2012.193 (2013). 22614017 PMC3484248

[b44] Luo J. H. . Transcriptomic and genomic analysis of human hepatocellular carcinomas and hepatoblastomas. Hepatology 44, 1012–1024, doi: 10.1002/hep.21328 (2006). 17006932 PMC1769554

[b45] Niinuma T. . Upregulation of miR-196a and HOTAIR drive malignant character in gastrointestinal stromal tumors. Cancer Res 72, 1126–1136, doi: 10.1158/0008-5472.CAN-11-1803 (2012). 22258453

[b46] Marquardt S. . Functional consequences of splicing of the antisense transcript COOLAIR on FLC transcription. Mol Cell 54, 156–165, doi: 10.1016/j.molcel.2014.03.026 (2014). 24725596 PMC3988885

[b47] Pelechano V. & Steinmetz L. M. Gene regulation by antisense transcription. Nature reviews. Genetics 14, 880–893, doi: 10.1038/nrg3594 (2013). 24217315

[b48] Wang E. . Predictive genomics: a cancer hallmark network framework for predicting tumor clinical phenotypes using genome sequencing data. Semin Cancer Biol 30, 4–12, doi: 10.1016/j.semcancer.2014.04.002 (2015). 24747696

[b49] Claudio J. O. . HACS1 encodes a novel SH3-SAM adaptor protein differentially expressed in normal and malignant hematopoietic cells. Oncogene 20, 5373–5377, doi: 10.1038/sj.onc.1204698 (2001). 11536050

[b50] Noll J. E. . SAMSN1 is a tumor suppressor gene in multiple myeloma. Neoplasia 16, 572–585, doi: 10.1016/j.neo.2014.07.002 (2014). 25117979 PMC4198825

[b51] Amend S. R. . Whole Genome Sequence of Multiple Myeloma-Prone C57BL/KaLwRij Mouse Strain Suggests the Origin of Disease Involves Multiple Cell Types. PloS one 10, e0127828, doi: 10.1371/journal.pone.0127828 (2015). 26020268 PMC4447437

[b52] Fukuda Y., Washio T. & Tomita M. Comparative study of overlapping genes in the genomes of Mycoplasma genitalium and Mycoplasma pneumoniae. Nucleic Acids Res 27, 1847–1853 (1999). 10101192 10.1093/nar/27.8.1847 PMC148392

[b53] Uesaka M. . Bidirectional promoters are the major source of gene activation-associated non-coding RNAs in mammals. BMC genomics 15, 35, doi: 10.1186/1471-2164-15-35 (2014). 24438357 PMC3898825

[b54] Fujita P. A. . The UCSC Genome Browser database: update 2011. Nucleic Acids Res 39, D876–882, doi: 10.1093/nar/gkq963 (2011). 20959295 PMC3242726

[b55] Sonnhammer E. L. & Ostlund G. InParanoid 8: orthology analysis between 273 proteomes, mostly eukaryotic. Nucleic Acids Res 43, D234–239, doi: 10.1093/nar/gku1203 (2015). 25429972 PMC4383983

[b56] Landgraf P. . A mammalian microRNA expression atlas based on small RNA library sequencing. Cell 129, 1401–1414, doi: 10.1016/j.cell.2007.04.040 (2007). 17604727 PMC2681231

[b57] Liao B. Y. & Zhang J. Evolutionary conservation of expression profiles between human and mouse orthologous genes. Molecular biology and evolution 23, 530–540, doi: 10.1093/molbev/msj054 (2006). 16280543

