# Supplementary material for: The Evolution and Expression Pattern of Human Overlapping lncRNA and Protein-coding Gene Pairs
Source: Sci Rep. 2017 Mar 27;7:42775. doi: 10.1038/srep42775 (PMC5366806; doi:10.1038/srep42775)
Supplement: Supplementary Information [file srep42775-s1.pdf]

The Evolution and Expression Pattern of Human Overlapping lncRNA and Protein-coding Gene Pairs

Qianqian Ning, Yixue Li, Zhen Wang, Songwen Zhou, Hong Sun and Guangjun Yu

Supplementary Methods

Correlation between members of lncRNA-coding pairs

In order to determine the expression correlation between members of lncRNA-coding pairs, we calculated the correlation used the Spearman correlation coefficient and Pearson correlation coefficient. Also the non-strand-specific and strand-specific expression data were used to test the correlation. All these metrics showed similar correlation patterns, therefore, we chose the Spearman correlation using strand-specific data for further analyses. In normal samples, the correlation was computed for each lncRNA-coding pair across tissues using the mean expression level of genes and across diseases in cancer samples. The statistical significance of each correlation was corrected for multiple hypotheses testing using the Hochberg's procedure. The correlation of random lncRNA-coding pairs was computed as above.

Statistical analysis

The statistical analyses including GO analysis was calculated and figures were drawn using the R programming language.

Supplementary Tables:

Supplementary Table 1: Preference of overlap in old group of protein-coding genes.

Age (Myr)	Overlap with lncRNA genes		Non-overlapping		Total
	Obs. (%)	Exp.	Obs.	Exp.	
0~90	239 (11.2)	553.8	1901	1596.2	2140
90~300	573 (17.1)	866.7	2776	2482.3	3349
>300	4235 (30.2)	3626.5	9778	10386.5	14013
Total	5047 (25.9)		14455		19502

Chi square test was used to test for statistical significance: $\chi^2 = 513.5$, p value $< 2.2\times10^{-16}$. The percentage in the parenthesis was calculated as the number of genes in lncRNA-coding pairs divided by the total number of genes in each age group.

Supplementary Table 2: The evolutionary age of genes in human lncRNA-coding pairs.

		lncRNA genes in lncRNA-coding pairs										
	Species	Human	Chimp	Gorilla	Orangutan	Macaque	Mouse	Opossum	Platypus	Chicken	*Xenopus*	Total
	Evolutionary age (Myr)	0	6	7	14	25	90	180	200	300	370	
Protein-coding genes in pairs	Human[1]	65	1	0	2	27	31	9	12	10	2	159
	Chimp[1]	8	0	0	0	1	1	0	1	0	0	11
	Gorilla[1]	9	0	1	0	8	2	3	1	0	0	24
	Orangutan[1]	27	0	0	1	12	5	1	2	2	2	52
	Macaque[1]	62	0	1	0	18	12	1	1	1	1	97
	Mouse[1]	71	0	0	2	30	36	3	4	3	6	155
	Opossum[1]	109	1	2	7	57	68	23	14	12	10	303
	Platypus[1]	126	0	1	5	47	84	20	31	6	7	327
	Chicken[1]	245	1	3	12	206	127	49	49	88	39	819
	Xenopus[1]	1807	4	9	54	1117	983	418	412	577	548	5929
	Total	2529	7	17	83	1523	1349	527	527	699	615	7876

[1] the species containing human orthologous genes that was farthest away from human, according to the phylogenetic distribution of the species. The values in this matrix is the number of lncRNA-coding pairs that protein-coding and lncRNAs originated from corresponding time period.

Supplementary Table 3: Composition of overlapping configurations of lncRNA-coding pairs.

**Pattern**	**OEB**	**T2T**	**H2H**	**SEB**	**H2T**	**Total**	**P value[1]**
Conserved	348 (71.5)	49 (10.0)	72 (14.8)	17 (3.5)	1 (0.2)	487	0.05
Non-conserved	5230	707	944	426	82	7389	
Total	5578 (70.8)	756 (9.6)	1016 (12.9)	443 (5.6)	83 (1.1)	7876	

[1] the p value was tested by comparison of the proportions of overlapping configurations between the conserved and total pairs. The number in the parenthesis was the percentage of lncRNA-coding pairs with the overlapping configuration in corresponding row.

Supplementary Table 4: Promotion of the alternative splicing of protein-coding genes in lncRNA-coding pairs.

**Coding gene**	**Overlap with lncRNA**		**Non-overlapping**		**Total**
	Obs. (%)	Exp.	Obs.	Exp.	
S	651 (15.6)	1148.3	3515	3017.7	4166
M	5641 (30.2)	5143.7	13021	13518.3	18662
Total	6292		16536		22828
**LncRNA**	**Overlap with coding**		**Non-overlapping**		**Total**
	Obs. (%)	Exp.	Obs.	Exp.	
S	5312 (46.5)	5362..7	6110	6059.3	11422
M	1978 (48.2)	1927.3	2127	2177.7	4105 (26.4)[1]
Total	7290		8237		15527

[1] The number in the parenthesis is the percentage of lncRNA genes with multiple transcripts in all lncRNA genes.

As for the rows, **S** stands for the genes have only one product and **M** for at least two products. The percentage in the parenthesis was calculated as the number of genes in lncRNA-coding pairs divided by the total number of genes in each alternative splicing group. Chi square test was used to test the significance of the 2×2 contingency table of coding genes: χ^2 = 362.9, p value < 2.2×10^{-16} and lncRNA genes: χ^2 = 3.3, p value = 0.07.

Supplementary Table 5: The preference of overlapping configurations for the alternative splicing pattern.

	**OEB**		**T2T**		**H2H**		**SEB**		**H2T**		**Total**
	**Obs.**	**Exp.**	**Obs.**	**Exp.**	**Obs.**	**Exp.**	**Obs.**	**Exp.**	**Obs.**	**Exp.**	
SS	199	220.4	51	61.5	64	76.0	107	75.0	40	28.0	461
SM	3025	2701.5	543	753.2	639	931.9	1179	919.6	264	343.7	5650
MS	169	160.2	30	44.7	56	55.3	45	54.5	35	20.4	335
MM	796	1106.9	544	308.6	686	381.8	95	376.8	194	140.8	2315
Total	4189		1168		1445		1426		533		8761

As for the rows, **S** stands for one single product and **M** for more than one product from the gene; the first letter was representative for the lncRNA gene and the second for the protein-coding gene. Chi square test was used to test for statistical significance: χ^2 = 1971.8, p value < 2.2×10^{-16}.

Supplementary Table 6: The proportion of lncRNA-coding pairs containing identical TFBS(s) in different-strand overlaps.

**Configuration**	**Identical TFBS (%)**	**No identical TFBS**	**Total**
OEB	243 (4.36)	5335	5578
T2T	32 (4.23)	724	756
H2H	99 (9.74)	917	1016
Total	347 (5.1)	6976	7350

The promoter region was defined as 1Kb upstream of the transcriptional start site. Chi square test was used to test for statistical significance: χ^2=52.9, p value=3.2×10^{-12}.

Supplementary Figures:

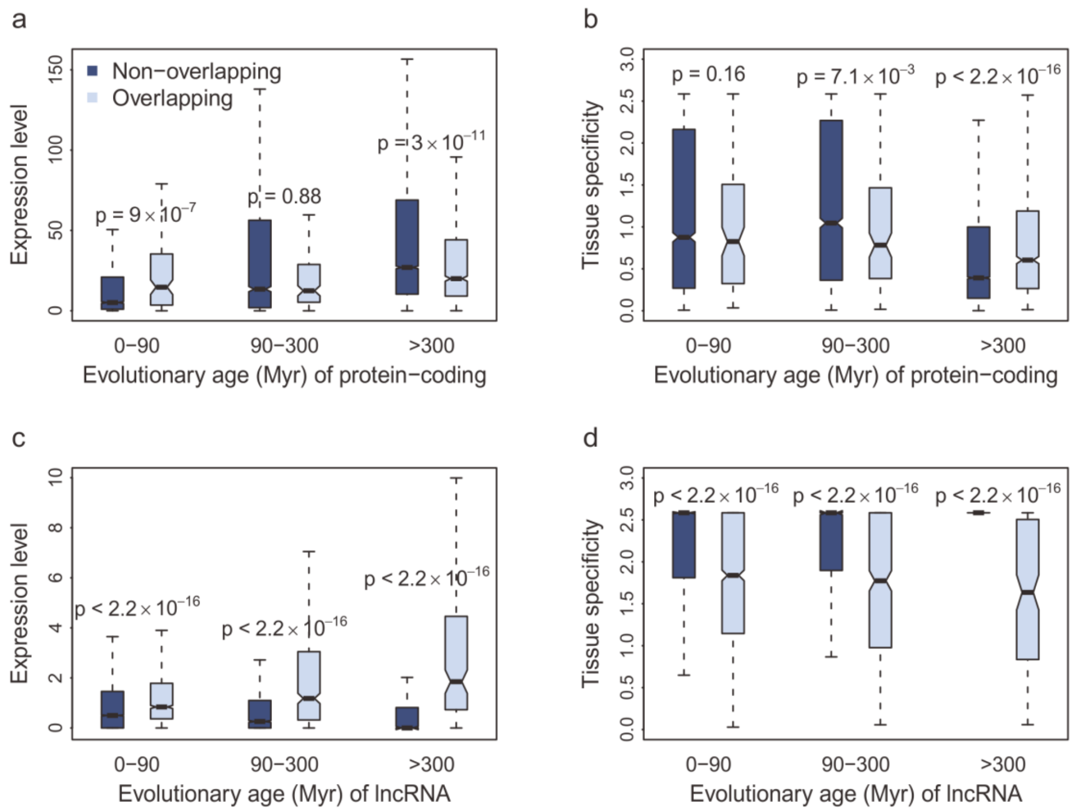

Supplementary Fig. 1: The effect of overlap on the expression of chimp lncRNA-coding pairs. (**a, c**) The maximum expression level (RPKM) of protein-coding (a) or lncRNA (c) genes by evolutionary age. (**b, d**) The tissue specificity of protein-coding (b) or lncRNA (d) genes by evolutionary age.

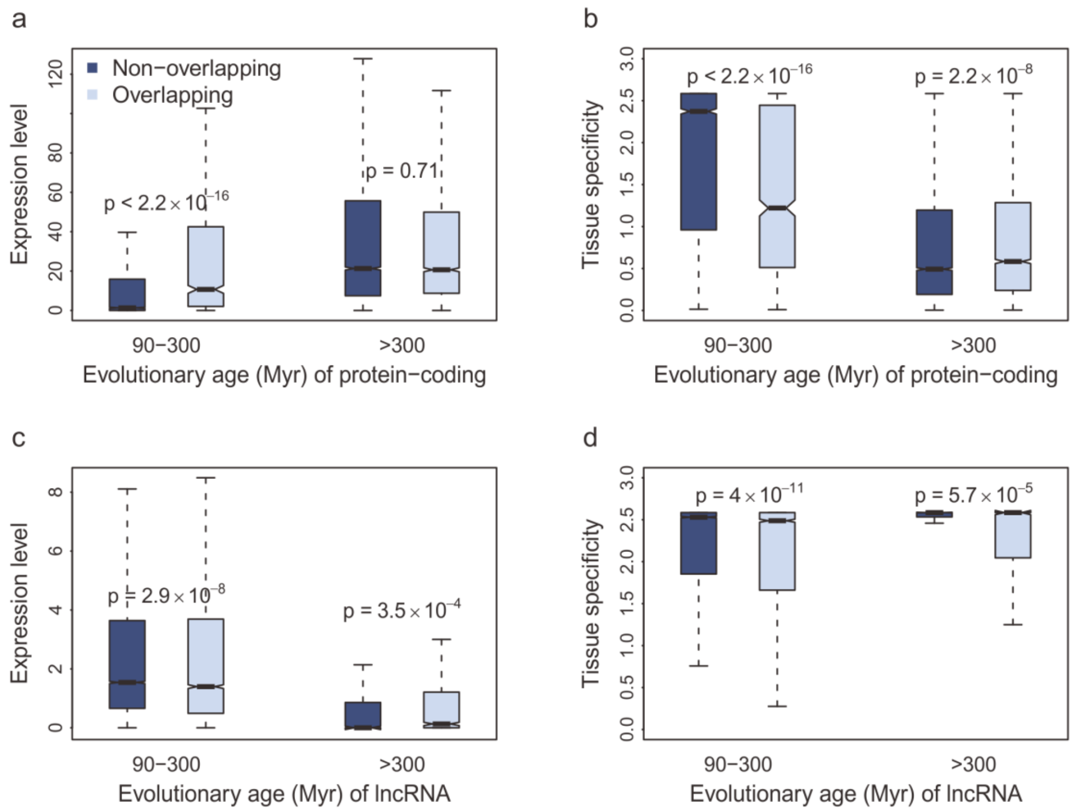

Supplementary Fig. 2: The effect of overlap on the expression of mouse lncRNA-coding pairs. (**a, c**) The maximum expression level (RPKM) of protein-coding (a) or lncRNA (c) genes by evolutionary age. (**b, d**) The tissue specificity of protein-coding (b) or lncRNA (d) genes by evolutionary age.

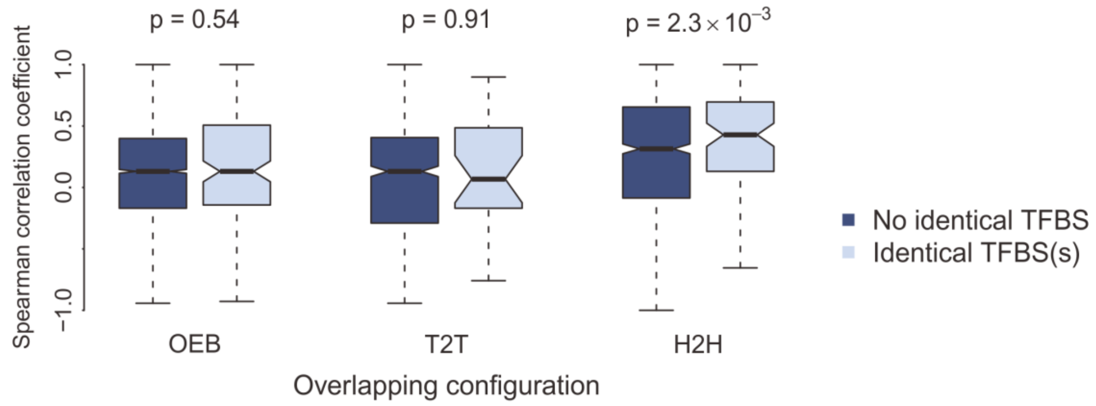

Supplementary Fig. 3: The effect of the similar regulatory sequence on the Spearman correlation coefficient of lncRNA-coding pairs by overlapping configuration.

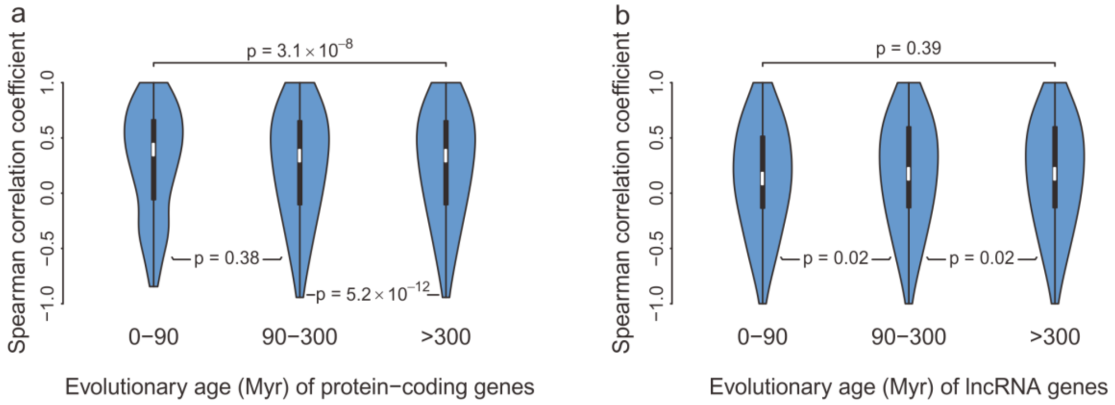

Supplementary Fig. 4: The age-specific expression correlation of lncRNA-coding pairs. (a, b) Distribution of Spearman correlation coefficients of lncRNA-coding pairs by evolutionary age of protein-coding (a) or lncRNA (**b**) genes.

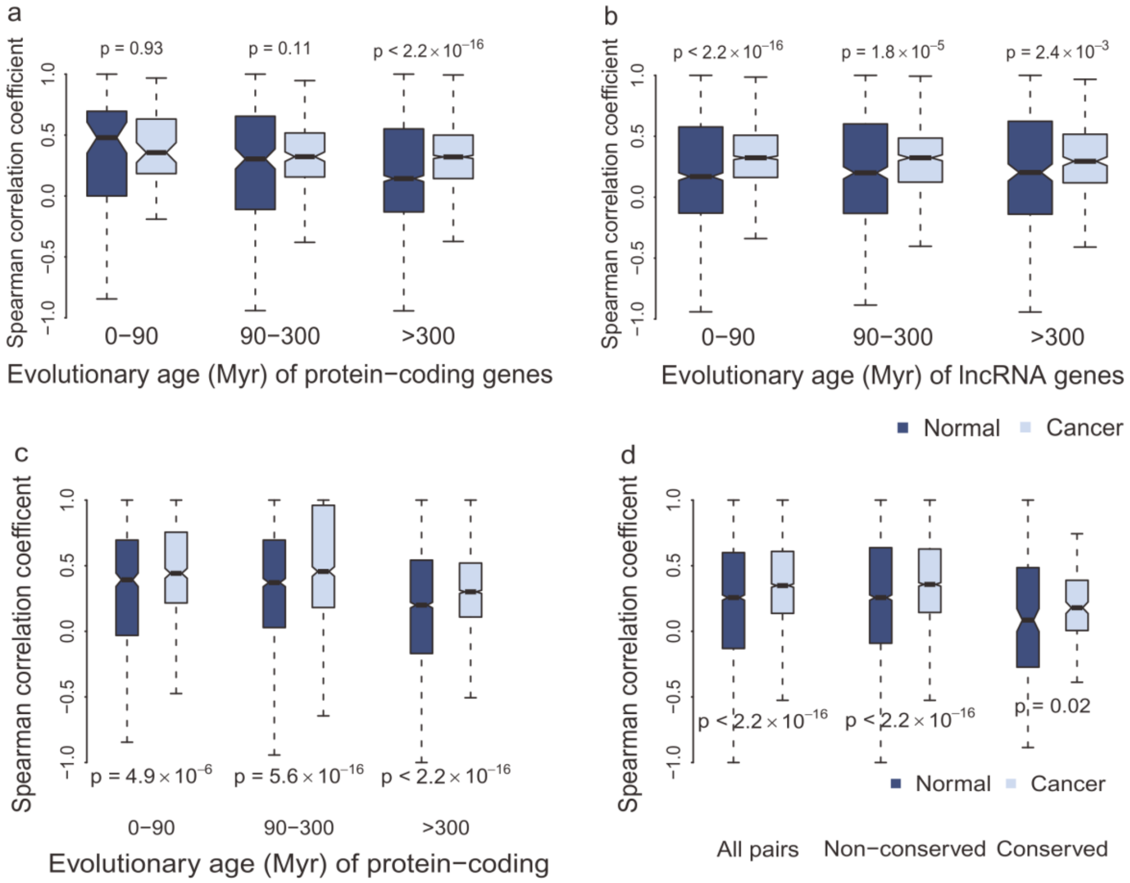

Supplementary Fig. 5: The expression correlation of overlapping genes in normal and cancer. (a, b) Boxplot of Spearman correlation coefficient of lncRNA-coding pairs by evolutionary age of protein-coding (A) or lncRNA (**B**) genes. (c) Boxplot of Spearman correlation coefficient of coding-coding pairs by evolutionary age of protein-coding genes. Protein-coding genes were integrated into three age groups as lncRNA genes. For each age group of protein-coding genes, any coding-coding pair including the genes in a group was

clustered into the corresponding age group of coding-coding pair.

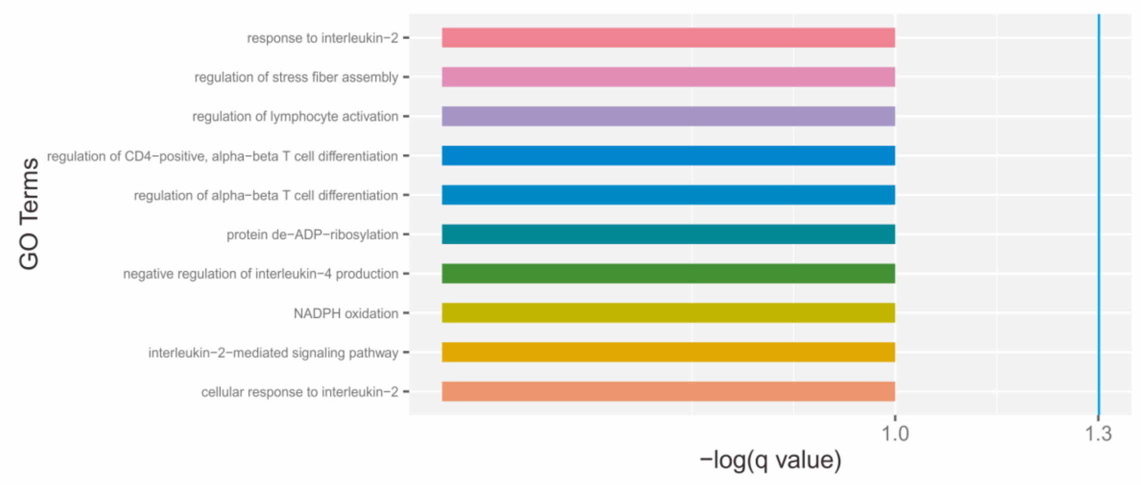

Supplementary Fig. 6: GO enrichment for relative younger protein-coding genes in non-conserved pairs. Only the top 10 terms were displayed and the term with values greater than 1.3 was significant. The q value was the p value adjusted by FDR and this gene set included 276 genes with evolutionary age younger than 300 Myr.

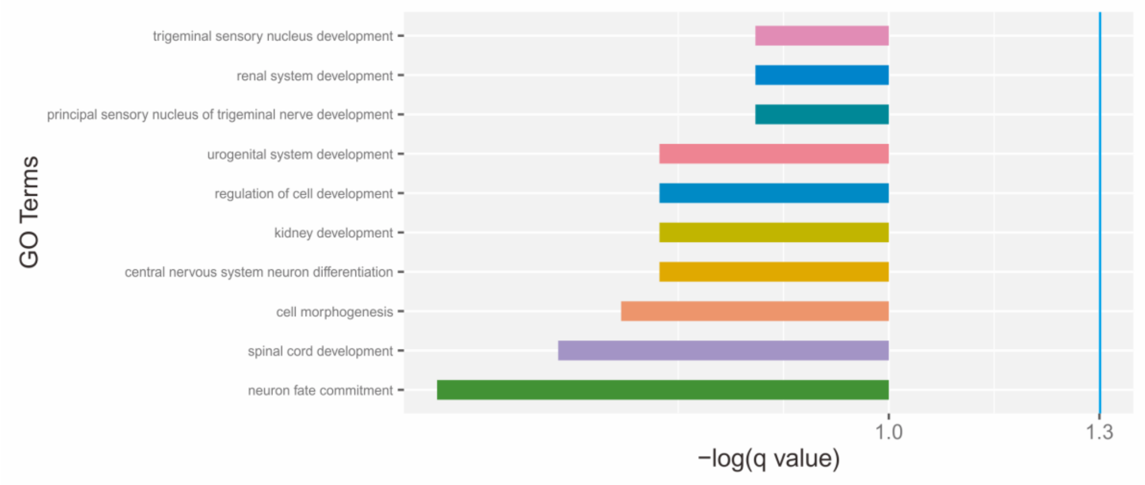

Supplementary Fig. 7: GO enrichment for protein-coding genes in conserved pairs. Only the top 10 terms were displayed and the term with values greater than 1.3 was significant. The q value was the p value adjusted by FDR and this gene set included 194 genes.

Supplementary Files:

Supplementary File 1: 487 lncRNA-coding pairs conserved among human, chimp and mouse.

Supplementary File 2: All lncRNA-coding pairs and blocks.

Supplementary File 3: All lncRNA-coding pairs left for analysis in cancer.

Supplementary File 4: lncRNA-coding pairs negatively correlated in normal and positively correlated in cancer.

Supplementary File 5: The GO significant categories for old protein-coding genes in non-conserved pairs.
